# Regulation of regulatory T cells and tumor‐associated macrophages in gastric cancer tumor microenvironment

**DOI:** 10.1002/cam4.6959

**Published:** 2024-02-04

**Authors:** Zhang Shaopeng, Zheng Yang, Fang Yuan, Huang Chen, Qiu Zhengjun

**Affiliations:** ^1^ Department of Gastrointestinal Surgery, Shanghai General Hospital Shanghai Jiaotong University School of Medicine Shanghai China

**Keywords:** gastric cancer, Hp, TAMs, Tregs, tumor microenvironment

## Abstract

**Introduction:**

Despite advancements in the methods for prevention and early diagnosis of gastric cancer (GC), GC continues to be the fifth in incidence among major cancers and the third most common cause of cancer‐related death. The therapeutic effects of surgery and drug treatment are still unsatisfied and show notable differences according to the tumor microenvironment (TME) of GC.

**Methods:**

Through screening Pubmed, Embase, and Web of Science, we identified and summarized the content of recent studies that focus on the investigation of *Helicobacter pylori* (Hp) infection, regulatory *T* cells (Tregs), and tumor‐associated macrophages (TAMs) in the TME of GC. Furthermore, we searched and outlined the clinical research progress of various targeted drugs in GC treatment including CTLA‐4, PD‐1\PD‐L1, and VEGF/VEGFR.

**Results:**

In this review, the findings indicate that Hp infection causes local inflammation and leads to immunosuppressive environment. High Tregs infiltration in the TME of GC is associated with increased induction and recruitment; the exact function of infiltrated Tregs in GC was also affected by phenotypes and immunosuppressive molecules. TAMs promote the development and metastasis of tumors, the induction, recruitment, and function of TAMs in the TME of gastric cancer are also regulated by various factors.

**Conclusion:**

Discussing the distinct tumor immune microenvironment (TIME) of GC can deepen our understanding on the mechanism of cancer immune evasion, invasion, and metastasis, help us to reduce the incidence of GC, and guide the innovation of new therapeutic targets for GC eventually.

## 1 INTRODUCTION

Although therapeutic methods have been developed, gastric cancer (GC) still ranks as the third leading cause of cancer death and has a high incidence and fatality rate worldwide.^1^ The tumor microenvironment (TME) is considered to be strongly linked to the efficacy of antitumor treatment. TME is rather complex and very different from the normal tissue which has gained increasing attention over the past decades and it is crucial to comprehend the precise biology of the TME. During the recruitment of tumor‐associated signals, various immune cell components including endothelial cells, fibroblasts, macrophages, and lymphocytes infiltrate the tumor immune microenvironment.^2^ As a distinct influencing factor in GC, *Helicobacter pylori* (Hp) infection was proved to reprogram TME through secreting multiple cytokines which can affect immune cells differentiation and migration. Tumor‐associated macrophages (TAMs) and regulatory T cells (Tregs) are two main immunosuppressive cells in TME, TAMs were tightly related to the angiogenesis proliferation and anti‐apoptosis in supporting of tumor growth and metastasis.^3^ Accumulation of Tregs in TME was considered to be related with poor prognosis due to its function on suppressing CD8^+^ T cells but the exact mechanism remains to be confirmed.^4^ In addition, many other elements are involved. Tumor‐infiltrating lymphocytes (TILs) such as CD4^+^ T cells, CD8^+^ T cells, innate immune cells including dendritic cells (DCs) and natural killer cells (NKs) are known to have a decisive impact on the characteristic of TME in GC.^5^ CD4^+^ T cells contribute to the regulation of immune responses by influencing the activation of CD8^+^ T cells which recognize tumor antigens and execute cytotoxic functions. DCs capture tumor antigens, process them, and present antigenic peptides to CD4^+^ and CD8^+^ T cells while NK cells play a role in immunosurveillance by directly killing tumor cells and producing cytokines. Meanwhile, gastric cancer tissue‐derived mesenchymal stem cells (GC‐MSCs) was proved to possess the ability to transfer into cancer‐associated fibroblasts (CAFs) under the stimulation of IL‐6 and TNF‐α which promote tumor progression.^6^ Furthermore, CAFs can impair the balance of immunity homeostasis in TME through limiting the mobilization of effector cells while increasing the proportion of M2 and Tregs which further suppress the supervision and attack on tumor cells.^7^ Understanding the intricate interactions and regulatory mechanisms of these immune cell subtypes in TME is essential for developing effective immunotherapeutic strategies against GC.

In this review, we mainly introduced the influence of Hp infection in GC TIME briefly, at the same time, the regulation and function of Tregs, TAMs and related immune cytokines which interact closely with each other were reviewed. We hope this review may provide help for the design of novel therapy approaches for personalized treatment.

## 2 HP INFECTION CAUSES IMMUNOSUPPRESSIVE LOCAL ENVIRONMENT

**FIGURE 1:**
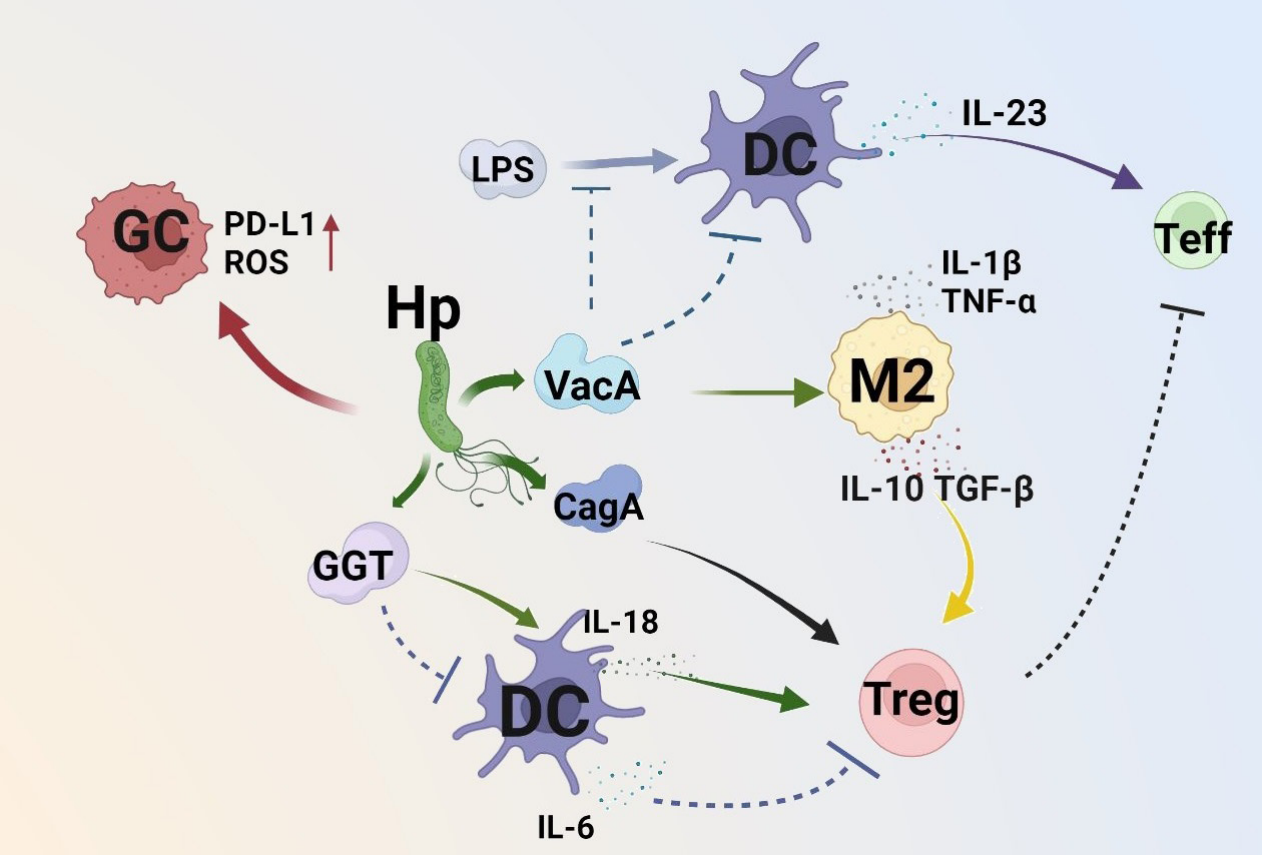
Hp infection establishs an immunosuppressive environment. Upon VacA effect, M2 cells can induce differentiation of Tregs by producing TGF‐β and IL‐10, VacA also inhibit T‐cell activation by suppressing maturation and IL‐23 secretion of DCs. Furthermore, GGT boosted DCs' ability to induce the expression of Foxp3 by regulation of IL‐18 and IL‐6. Additionally, Hp induced PD‐L1 expression and ROS production in GCs.

Hp infection, as an important carcinogen, provides a prerequisite for the occurrence of GC by reshaping local gastric microenvironment. Vacuolating cytotoxin A (VacA), γ‐glutamyl transpeptidase (GGT), and cytokines are main virulence determinants of Hp that contribute to the establishment of suppressive immune environment.^8^, ^9^, ^10^ VacA are secreted and act on T cells to inhibit T‐cell activation by suppressing IL‐23 secretion of CD11b + DCs and inducing IL‐10 and TGF‐β secretion in macrophages.^11^ Mathias et al. found that DCs were driven by Hp infection to transfer to tolerogenic phenotype, targeted deletion of the GGT and VacA genes impaired Hp's capacity to tolerize DCs.^12^ Meanwhile, Hp infection resulted in a milieu that promoted Tregs differentiation and expansion in a VacA‐dependent manner, followed by promoted gastric resident Tregs response which eventually contributed to immune escape. Eradication of Hp resulted in the down regulation of Tregs.^11^, ^13^ Accordingly, John Y et al. demonstrated that the Tregs/Th17 balance in VacA mutant was similar to WT Hp strain, and IL‐10 and TGF‐β neutralization reversed the Treg‐skewed response influenced by Hp infection.^14^ GGT promoted the generation of glutamate then active the glutamate receptors on DCs. On the one hand, it suppressed the release of IL‐6 and other pro‐inflammatory cytokines by DCs. On the other hand, GGT boosted DCs' ability to induce the expression of Foxp3 and the secretion of regulatory cytokines in co‐cultured naive T cells, especially in neonatally Hp infected mice.^8^ Under the regulation of Hp, gastric cancer stem cells can act as regulator of Th17/Treg ratio, Treg IL‐17^−^ lymphocytes can be transferred to IL‐17^+^ phenotype while Th17 Foxp3^−^ lymphocytes can become to Foxp3^+^ Tregs.^15^, ^16^ In brief, Hp infection causes local inflammation and leads to immunosuppressive environment, which result from the negative immune regulation described above (as shown in Figure 1). Followed by superficial gastritis (SG), chronic atrophic gastritis (CAG), intestinal metaplasia (IM), dysplasia and leading to GC. Therefore, understanding the mechanism of immune tolerance caused by Hp provides new clues for GC immunotherapy.

## 3 ROLE OF TREGS IN MICROENVIRONMENT OF GC

### 3.1 Increased Tregs in GC

Tregs are immunosuppressive lymphocytes who exert negative immunoregulatory effects by suppressing effector T cells (Teffs). The frequency of Tregs is increased in GC patients, and the proportion of Tregs in tumor tissue is the highest followed by tumor draining lymph nodes, ascites fluid, peripheral blood, and healthy control.^17^ Increased Tregs in the TME of GC can be roughly divided into two reasons: increasing inductive differentiation and recruitment.

#### 3.1.1 Induction

Increased induction of Tregs in GC patients was widely reported. Immune cytokines, such as TGF‐βand IL‐10 has been considered to play an indispensable role in the induction and function of Tregs. Consistent with it, the expression of TGF‐β and IL‐10 in GC patients was increased in blood samples compared to healthy donors. Coculture assay was further performed to demonstrate that gastric cancer cells promote the differentiation of Tregs through TGF‐β.^18^ As described above, Tregs are expressed at significantly higher levels in HP infected patients than in non‐infected patients, excessive IL‐10 and TGF‐β produced by gastric epithelial cells after HP infection are considered to responsible for it.^19^ Other studies indicates that Indoleamine 2, 3‐dioxygenase (IDO) is overexpressed in Hp‐infected gastric mucosa and promote Tregs differentiation whereas decreases the number of Th1, Th17, and Th12.^20^ In addition, many other mechanisms have been revealed to involved in the ascending induction of Tregs in GC TME. Hypoxia and low‐glucose condition are common features of solid tumors microenvironment, which have been thought to play key role in tumor‐mediated immune suppression by boosting the quantity and suppressive properties of Tregs.^21^, ^22^ Shogo Kumagai and his associates discovered that the increased generation of free fatty acids (FFAs) due to the activation of PI3K‐AKT signaling pathway promoted Tregs accumulation in the low‐glucose TME. Tumor‐infiltrating Tregs show stronger ability to use FFAs than other T‐cell subsets, thus they were more proliferative and less apoptotic in response to high concentration of FFAs under a low‐glucose condition.^23^ Hypoxia inducible factor‐1α (HIF‐1α) was favorably correlated with the depth of tumor invasion, lymph node metastasis and advancing tumor stage. For gastric cancer, the majority of Tregs were found nearby cancer cells that were HIF‐1‐expressing, and the increased level of TGF‐β derived from tumor cells under hypoxia condition was proved to promote the differentiation of intratumoral Tregs in GC TME.^24^ Further investigation showed that the accumulation of Tregs in GC may also correlated with cyclooxygenase‐2 (COX‐2) and prostaglandin E2 (PGE2). Tregs could produce a great amount of COX‐2, which stimulates the creation of PGE2 and induces Foxp3 expression in turn.^17^ Lin Ret al. found that bone marrow mesenchymal stem cells cause an increased production of IL‐10 and IFN‐γ in Hp‐induced GC, and correspondingly increase the ratio of Treg/Th17 to create an immunosuppressive in GC TME.^25^ Meanwhile, GC‐MSCs was proved to mediate antitumor immune response through promoting Tregs differentiation by releasing TGF‐β, IL‐6 and IL‐10 while inhibiting Th17 cells proliferation by PGE2 and IFN‐γ. It may explain why the ratio of Th17/Tregs was shown to be high in early disease, but only the increased accumulation of Tregs continue in advanced disease, and the infiltration of Th17 cells decrease gradually in the progress of disease.^26^ These results clearly imply that the growth and accumulation of Tregs in the GC are partly caused by tumor‐related mechanisms.

#### 3.1.2 Recruitment

The role of chemotactic factors in recruiting Tregs to the tumor site plays the most significant role in their accumulation. Gastric tumor infiltrated Tregs exhibit a higher affinity for CCL17/22 than effector T cells, and the level of chemokines CCL17/22 produced by DC cells in gastric tumors was significantly higher than normal gastric mucosa which induce the migration of Tregs in peripheral sites via CCR4 (the receptor for CCL17/22). CD8^+^ T cell secret higher levels of CCL22 and interferon‐γ (IFN‐γ) to increase PD‐L1 and IDO in Tregs which result in the accumulation of Tregs in cancer cells, and create a favorable environment for tumor growth eventually.^27^, ^28^ More than 70% of GC patients have been shown to have Hp infections that encourage the activation of Wnt/β‐catenin signaling and CCL28 has been shown to be a direct transcriptional target gene of β‐catenin in T cells. Increased expression of CCL28 regulated by β‐catenin in gastric cancer cells were responsible for the recruit and accumulation of Tregs in GC TME.^29^, ^30^ According to earlier research, the majority of Tregs express lymphoid homing receptors CD62L and CCR8, combination of other chemokines such as CCL22, CCL1 which causes a progressive rise in Treg numbers in tumors during the disease progresses.^31^ Jeong and colleagues examined the structural and histological characteristics of GC in superficial layers and deeper layers, they discovered that Tregs tended to gather in the deep layers due to the increased production of CCL2 in stromal cells, contributing to the formation of suppressive immune microenvironment in deeper layer and result in poor survival in diffuse type GC.^32^ Furthermore, the proliferate ability of Tregs was higher than CD4^+^Foxp3^−^ T cells in gastric tumor mucosa, which may potentially explain the enrichment of Tregs, but these local Tregs displayed a suppressive cytokine profile defined by high IL‐10, low TGF‐βand IFN‐γ production.^33^ Forkhead box protein M1 (FoxM1) plays a significant role in the development of cancer, a significant association between FoxM1 and Tregs in GC peripheral blood was confirmed recently, FoxM1 may participate in the process of immune escape in GC through recruiting Tregs via PI3K‐Akt‐FoxO signaling axis.^34^

### 3.2 Function of Tregs in GC

Tregs plays an important role in mediating immune TME in GC, more and more researches suggested that the function of Tregs in GC TME was not only affected by numbers but also related to phenotypes and immune molecules.

#### 3.2.1 Phenotypes

TCR‐inducible costimulatory receptor (ICOS), a member of the CD28 superfamily is known to induce the secretion of IL‐10 and TGF‐β. Higher ICOS^+^ Tregs are found in patients with advanced GC and patients with lower ICOS^+^ Tregs showed longer relapse‐free survival. Furthermore, ICOS^+^ Tregs was significantly higher in patients with the *H. pylori* antibody. Tumor necrosis factor (TNF)‐α/TNF receptor superfamily member 1B (TNFR2) was used to identify a new population of Tregs, TNF‐α/TNFR2 pathway can increase Foxp3 levels and latent TGF‐βproduction, The level of TNFR2^+^ Tregs in GC patients was closely correlated with both the TNM stage and N stage. TNFR2^+^Tregs show enhanced function and can serves as an independent risk factor for their prognosis. Tim‐3 was identified as an activation and terminal differentiation marker, Tim‐3^+^ Tregs secreted higher IL‐10 and TGF‐βthan Tim‐3^−^Tregs. In GC patients, high Tim‐3 expression on Tregs was associated with worse prognosis.

#### 3.2.2 Immune molecules

The role of Tregs is intricately connected to the expression of various immune checkpoint molecules and cell surface markers. Ongoing efforts are focused on the development of targeted drugs to influence these processes. Principal immune checkpoint targets encompass cytotoxic T lymphocyte antigen‐4 (CTLA‐4), programmed cell death 1 (PD‐1), and its ligand PD‐L1.The targeting drugs are currently being applied in clinical practice or are undergoing clinical trials, as indicated in Table 1. CTLA‐4 primarily governs the interaction between conventional T cells (Tconvs) and CD80 and CD86 molecules on antigen‐presenting cells (APCs). Through this mechanism, Tregs inhibit Tconvs by limiting CD28 co‐stimulation, inducing a state of diminished responsiveness known as anergy.^35^ Anti‐CTLA‐4 antibodies have a wide‐ranging impact by enhancing CD28 co‐stimulation in Tconvs. An innovative humanized anti‐CTLA‐4 variant, designed to be more compact and optimized for Fc‐mediated antibody‐dependent cellular cytotoxicity (ADCC), has demonstrated enhanced tumor penetration and a more potent tumor immune response in comparison to conventional anti‐CTLA‐4 therapies.^36^ In a similar vein, the PD‐L1/PD‐1 signaling pathway also hampers Tconvs activity, and blocking PD‐1 results in increased Tconvs activation. The combination of anti‐CTLA‐4 and anti‐PD‐1 treatments exhibits a synergistic effect and has received approval for the treatment of specific aggressive cancers.^37^ Other strategies designed to target Tregs involve molecules like TIM‐3,^38^ V‐domain Ig suppressor of T‐cell activation (VISTA),^39^ glucocorticoid‐induced TNFR‐related (GITR) protein agonistic antibody.^40^ These approaches have yielded promising results, and some combinations with immune checkpoint inhibitors are presently undergoing investigation in ongoing clinical trials for cancer therapy. Cell surface markers, such as CD25, CD39, and CD73, play pivotal roles in Treg function. Tregs expressing CD25 can outcompete Tconvs for IL‐2, but endeavors to reduce tumors in mice by targeting CD25 have shown limited success due to insufficient reduction of tumor‐infiltrating Tregs and disruption of IL‐2 receptor signaling in Tconvs.^41^ Encouragingly, CD39‐deficient and CD73‐deficient Tregs have demonstrated the ability to stimulate stronger antitumor immune responses in mice.^42^

**TABLE 1:** The list of clinical practice and trials about drugs target cytotoxic T lymphocyte antigen‐4 (CTLA‐4), programmed cell death 1 (PD‐1), and its ligand PD‐L1.

Target structure	Clinical trials	Study design	Research objects	Patient number	Groups	OS‐HR	PFS‐HR	Reference
PD‐1	NCT02872116	III	Previously untreated, unresectable, HER2 (−) G/GEJ/esophageal adenocarcinoma	1549	Nivolumab + chemo; chemo	0.80 (0.68–0.94)	0.77 (0.68–0.87)	[43]
	NCT02494583	III	Advanced/unresectable or metastatic G/GEJ C	763	Pembrolizumab; pembrolizumab + chemo; placebo + chemotherapy	Cps ≥ 1: Pembrolizumab versus placebo + chemo: 0.91 (0.69–1.18) Pembrolizumab + chemo versus placebo+ chemo: 0.85 (0.70–1.03)	Cps ≥ 1: Pembrolizumab versus placebo + chemo: 1.66 (1.37–2.01) Pembrolizumab + chemo versus placebo + chemo: 0.84 (0.70–1.02)	[44]
						Cps ≥ 10: Pembrolizumab versus placebo + chemo: 0.69 (0.49–0.97) Pembrolizumab + chemo versus placebo + chemo: 0.85 (0.62–1.17)	Cps ≥ 10: Pembrolizumab versus placebo + chemo: 1.10 (0.79–1.51)	
	NCT03745170	III	Untreated, unresectable locally advanced or metastatic G/GEJ C	650	Sintilimab + chemo; placebo + chemo	0.766 (0.626–0.936; *p* = 0.0090)	0.636 (0.525–0.771; *p* < 0.0001)	[45]
PD‐L1	NCT01772004	II	Unresectable, locally advanced or metastatic GC/GEJC	150	1 L‐mn (first‐line switch‐maintenance); 2L subgroup (second‐line)	–	–	[46]
	NCT02625610	III	Untreated, unresectable, HER2 (−), locally advanced or metastatic GC /GEJC	499	Avelumab; chemo	0.91 (0.74–1.11)	1.04 (0.85–1.28)	[47]
CTLA‐4	NCT03852251	Ib/II	Locally advanced/unresectable or metastatic locally advanced/unresectable or metastatic G/GEJ C	96	AK104 + chemo	–	–	[48]
	NCT02872116	III	Unresectable advanced or metastatic G/ GEJ Or esophageal C	3185	Nivolumab + chemo; nivolumab + ipilimumab	0.91 (0.77–1.07)	–	[49]
	NCT02340975	Ib/II	Metastatic or recurrent G/GEJ C	113	Second‐line: durvalumab + tremelimumab; durvalumab; tremelimumab; third‐line: durvalumab + tremelimumab; second‐line/third‐line D + T IFNγ+	–	–	[50]

**FIGURE 2:**
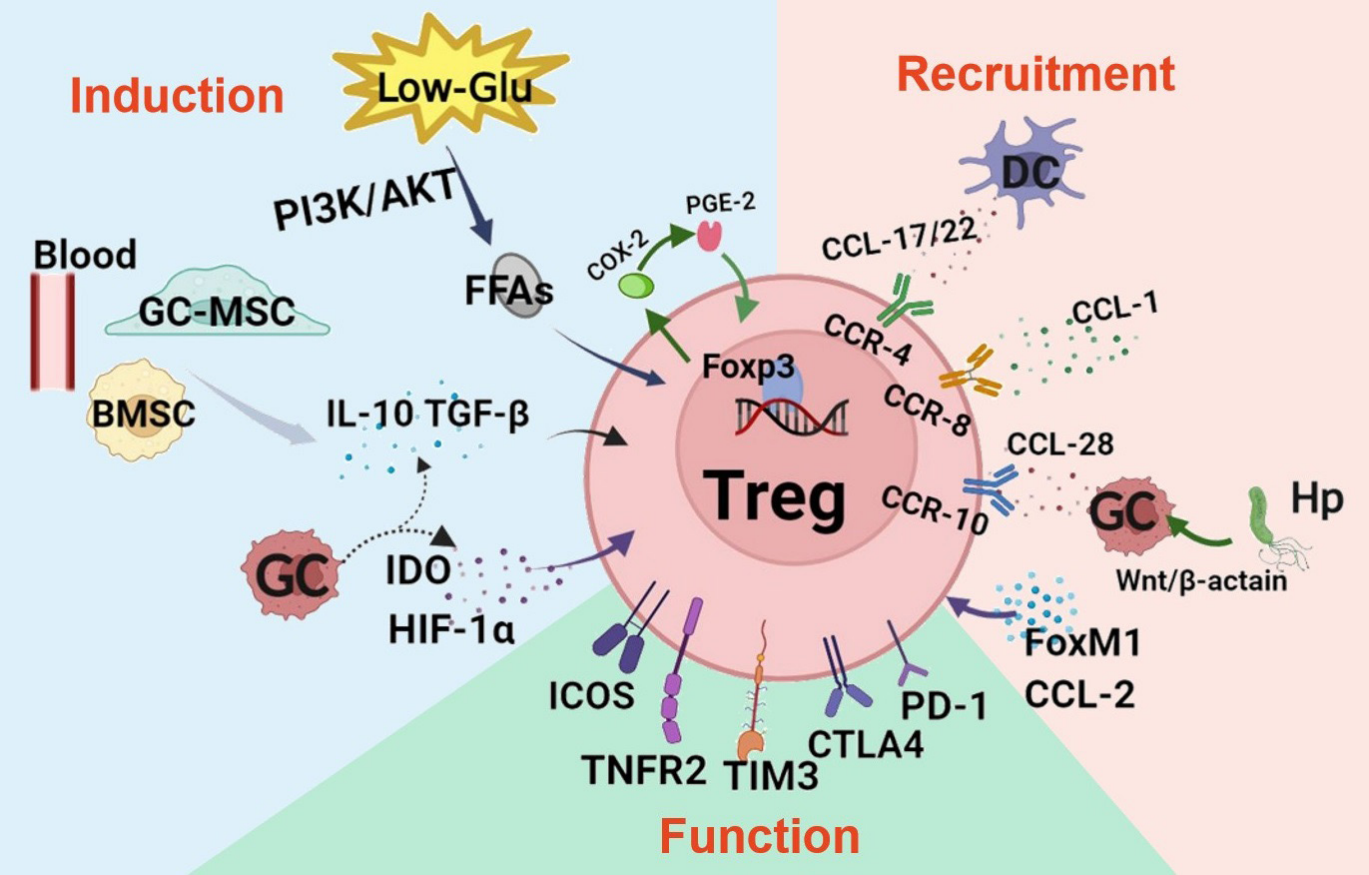
The changes of Tregs infiltrating in the microenvironment of GC can be roughly divided into: inducing differentiation and increasing recruitment. Under the low‐glu and hypoxia conditions in the tumor microenvironment, GC tumor cells may promote Treg differentiation by secreting FFAs, HIF‐1α, IDO, and other immunosuppressive factors including TGF‐β and IL‐10. DCs, and Hp can recruit Tregs in the GC microenvironment. CCL17, CCL22, CCL‐1, CCL‐2, CCL‐28, and FoxM1 can mediate the aggregation of Tregs. Additionally, Tregs exercise inhibitory function depending on the expression levels of ICOS, TNFR2, PD‐L1, CTLA‐4, TIM3, and distribution simultaneously.

In conclusion, increased recruitment and induction are the two primary causes of greater Treg infiltration in the TME of GC respectively. The exact function of infiltrated Tregs in GC was also affected by other factors like phenotypes and immunosuppressive molecules (as shown in Figure 2).

## 4 TAM IS A MAJOR DETERMINANT OF OUTCOME OF GC

### 4.1 Function of TAM in GC

Macrophage plays central role in innate and adoptive immune regulation, according to their anti and pro‐tumorigenic functions. The difference in metabolism between M1 and M2 partly responsible for their opposite immune functions, CD68 + CD86+ M1 are enriched in microsatellite instability (MSI) and intestinal subtype characterized by enhanced glycolysis and fatty acid synthesis while M2 exhibit decreased glycolysis and fatty acid oxidation.^51^ TAMs may promote the development and metastasis of tumors by secreting and releasing immune cytokines. For instance, TAM derived cytokines, such as IL‐6, IL‐1β, IL‐8, TNF‐α, CCL‐17, and CCL‐22 significantly contribute to proliferation of GC cells, immunosuppression and angiogenesis in TME through the mediation of immune surveillance which support tumor growth and metastasis as a result.^3^ The prevailing view points out that M2 accounts for a greater proportion of TAM than M1, nevertheless, M1 infiltration was found to be positively correlated in GC. Stimulator of interferon genes (STING) is a key component in the innate immune system, altered STING in macrophages was reported to promote the polarization of M1 and cause the apoptosis of GC cells via activated IL‐6R/JAK–STAT pathway.^52^

The function of M2 in GC has been deeply explored in the past decades. M2 exhibit functions through multiple ways. One important way is inducing tumor angiogenesis, increasing their VEGF secretion and resulting in the formation of neo‐angiogenic cells, therefore, several anti‐vascular endothelial growth factor (VEGF) and VGFR inhibitors have been explored for the therapy on GC.^53^ For example, Ramucirumab, a monoclonal selective VEGFR2 antibody has been observed to suppressive VEGFR2, which decreased M2 infiltration and inhibited the production of chemokine, which in turn delay GC tumor progress.^54^ In addition, many other researches are conducted to assess the value of VEGF/VEGFR‐targeted drugs in the treatment of GC as shown in Table 2. Furthermore, HIF transcription factors targeted several angiogenic factors including VEGF, M2 was reported to show higher level in regions of hypoxia such as GC tissue, the combination of these two factors result in enhanced angiogenesis in these hypoxia areas. Macrophages also boost the capacity of tumor cells to invasive blood vessels, and intravasation efficiency is directly correlated with the migration of tumor cells toward blood vessels. Fortunately, pharmaceutical suppression of epidermal growth factor receptor (EGFR) activation can prevent intravasation.^55^ In addition to the functions mentioned above, co‐culture with M2 also increases STAT3 activation and enhances phosphorylation of AKT and ERK1/2 in GC cells, leading to the proliferation and progression of cancer cells.^56^ The cross talk between Tregs and M2 was nonnegligible in TME, M2 was reported to contribute to an immunological suppressive phenotype through increasing Tregs differentiation by the synthesis of IL‐10 and TGF‐β and accelerating Treg recruitment by producing CCL22.^57^

**TABLE 2:** The list of clinical practice and trials about drugs target VEGF/VEGFR.

Target Structure	Clinical trials	Study design	Research objects	Patient number	Groups	OS‐HR	PFS‐HR	Reference
VEGFR	NCT00477711	II	Locally advanced or metastatic esophageal GC	47	Cetuximab + chemo	–	–	[58]
	NCT00824785	III	Locally advanced or metastatic esophageal GC	553	Chemo; chemo + panitumumab	1.28 (0.77–2.13)	1.30 (0.78–2.16)	[59]
	JapicCTI‐090849	II	Refractory, unresectable or recurrent GC	83	Nimotuzumab + irinotecan; irinotecan	0.994 (0.618–1.599)	0.860 (0.516–1.435)	[60]
VEGF/VEGFR	NCT00548548	III	Metastatic or unresectable locally advanced G/GEJ C	774	Bevacizumab; placebo	0.87 (0.73–1.03)	0.80 (0.68–0.93)	[61]
	Anzctr12612000239864	II	Metastatic or locally recurrent GC	152	Regorafenib; placebo	0.74 (0.51–1.08)	0.40 (0.28–0.59)	[62]
	NCT03333967		Advanced GC	737	Apatinib; apatinib + chemo	–	–	[63]

### 4.2 Induction of TAM in GC

The higher infiltration of M2 in GC was associated with several mechanisms. First, GC cells significantly contributed to the activation TAM in response to the pro‐inflammatory reaction, which in turn activated NF‐B and STAT3 pathways in cancer cells, leading to the development and progression of gastric cancer.^64^ Co‐culture with GC cells converted M1 into M2 that contribute to progression in GC with peritoneal dissemination, and macrophages also help to accelerate the process of peritoneal dissemination via activating EGFR signaling pathways. Second, Hp infection was reported to impair M1 macrophage response and promote M2 polarization recently. Third, the various levels of multiple cytokines are involved in the regulation of M2 enrichment in TME. Colony‐stimulating factor‐1 (CSF‐1)/CSF‐1 receptor signaling is essential for macrophage survival and transition from M0 into M2, and activated CSF‐1R signaling promotes differentiation, proliferation and chemotaxis of M2.^65^ As one of the major elements in tumor stroma, gastric cancer‐derived mesenchymal stromal cells (GC‐MSCs) was reported to trigger M2 polarization through activation of the JAK2/STAT3 signaling pathway via high secretion of IL‐6/IL‐8. Blocking IL‐6/IL‐8 through treatment of neutralizing antibodies reduced the rate of epithelial mesenchymal transition (EMT) in GC cells dramatically.^66^ Furthermore, IL‐6 expression was more pronounced in GC patients' tumor tissues than in normal tissues and positively associated with M2 activity. IL‐6 could promote the differentiation of IL‐10^high^TGF‐β^high^IL‐12^low^ M2 macrophages via the activation of STAT3 phosphorylation.

### 4.3 Recruitment of TAM in GC

Secretion of cytokines and chemokines by GC cells can attract M2 infiltration. For example, TGF‐β could elevate M2 proportion and linked to worse overall survival in GC. On the one hand, TGF‐β levels were increased significantly in the macrophages cocultured with GC cell lines, followed by the invasion‐related genes MMP9 and VEGF‐A expressions in GC cells were elevated due to activated TGF‐β/BMP signaling.^67^ On the other hand, transcription factor NF‐кB and TGF released by M2 could enhance the levels of Kindlin‐2 in GC cells which was discovered to promote the invasion of GC cells in vivo and strongly positively linked with TNM stage and worse overall survival.^68^ Monocytes respond strongly to CCL2, the chemoattractant which is produced by tumor cells and attracts monocytes to the tumor locations. In GC patients with peritoneal dispersion, Takahisa and his colleagues discovered a considerably larger number of peritoneal M2, the majority of which were CCR2 positive. Inhibition of CCL2–CCR2 axis is another strategy to prevent M2 migration, lessen tumor burden and reduce metastasis in experimental models.^69^, ^70^ New evidence indicated that Wnt/β‐catenin activation was essential for M2 infiltration, first, IL‐4/TGF‐β mediated M2 polarization by regulating c‐Myc.^71^ At the same time, Wnt5a also triggered Ca2+/CaKMII pathway in M2 and increasing CCL2 paracrine secretion, knockdown of β‐catenin in M2 inhibited TAMs' tumor‐promoting activities.^72^ In addition, M2 secret high levels of growth differentiation factor 15 (GDF15) to enhance the chemo‐resistance of GC cells by promoting fatty acids β‐oxidation.^73^ IL‐10 producing M2 was found to makes immune evasion easier in numerous cancers. GC patients with high IL‐10 producing M2 infiltration exhibited a worse therapeutic response and a worse survival to adjuvant chemotherapy, which was characterized by increased Treg infiltration and CD8^+^ T‐cell malfunction. Additionally, tumor intrinsic features such as EBV status, PD‐L1 expression, and genomic stability in GC were associated with IL‐10 producing M2 invasion.^74^ Further investigation on the signaling mechanisms that related to the differentiation and recruitment of macrophages in GC TME and how they facilitate tumor initiation and progression will lead to new insights into the evolution of the microenvironments, thereby providing potential targets for anticancer therapeutics.

**FIGURE 3:**
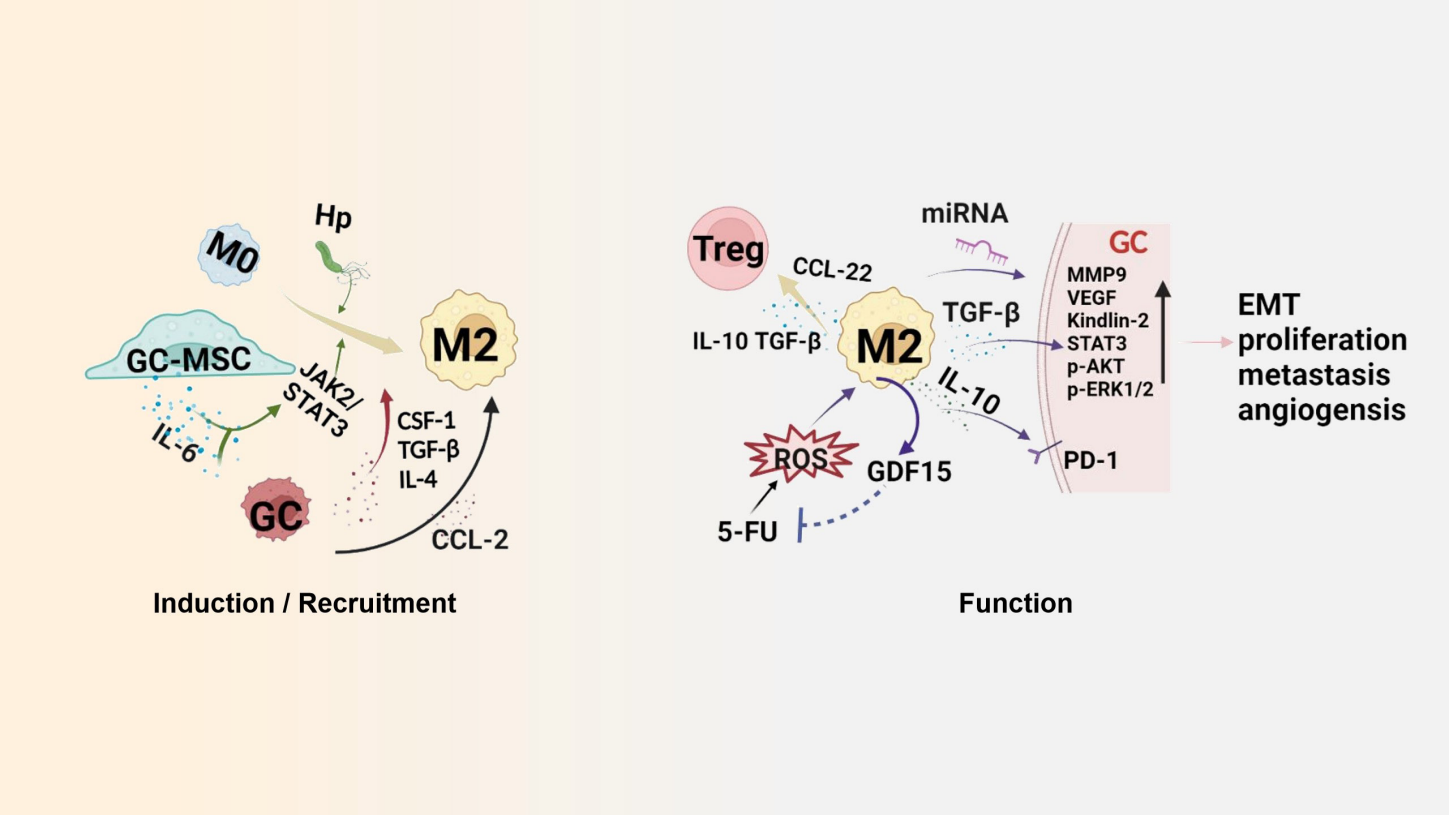
Variable mechanisms of M2 cells induction & recruitment and function in GC tumor microenvironment. Hp infection was reported to promote M2 polarization; furthermore, GC‐MSC and GC trigger M2 polarization through JAK2/STAT3 pathway via IL‐6 release. In addition, the secretion of IL‐4, TGF‐β, CSF‐1, and CCL‐2 by GC were proved to promote M2 polarization and recruitment. Function: M2 can accelerate GC EMT, proliferation, metastasis and angiogensis through secretion of TGF‐β, IL‐10 by enhancing expression of MMP9, VEGF, STAT3, and PD‐L1, and phosphorylation of AKT and ERK1/2. In addition, release of miRNA, GDF15, and CCL‐22 were related to the pro‐tumorigenic functions of M2 cells.

Briefly, M2 is a main portion of the infiltrated macrophages in solid tumors (as shown in Figure 3). Further investigations on the elucidation of its enrichment and function mechanisms in TME are essential for improving immunotherapy response in GC.

## 5 CONCLUSION

Chronic Hp infection induces gastric cancer depends on the regulation of immune response, such as inhibits T‐cell activation, promotes Tregs infiltration which results in immunosuppressive local environment. Levels of Tregs and M2 cells are increased in GC through increasing the differentiation and recruitment accompanied by fluctuated cytokines secretion which are considered as the most potential targets, and several trials are under development to ameliorate TME by restraining them to increase the efficacy of immune therapy. We hope this review can provide clues for further investigation about the understanding of the characteristics of TME in GC which help to predict therapy outcomes and design appropriate drugs for anti‐GC therapeutics.

## AUTHOR CONTRIBUTIONS

**Zhang Shaopeng:** Data curation (equal); writing – original draft (equal). **Zheng yang:** Software (equal); writing – original draft (equal). **Fang yuan:** Writing – review and editing (equal). **Huang Chen:** Writing – review and editing (equal). **Qiu zhengjun:** Funding acquisition (equal); project administration (equal); writing – review and editing (equal).

## FUNDING INFORMATION

This study was supported by grants from the National Natural Science Foundation of China (81974372, 82203751, and 82072662).

## CONFLICT OF INTEREST STATEMENT

None declared.

## Data Availability

The authors confirm that the data supporting the findings of this study are available within the article [and/or] its supplementary materials.
